# Vertical tumor-positive resection margins and the risk of residual neoplasia after endoscopic resection of Barrett’s neoplasia: a nationwide cohort with pathology reassessment

**DOI:** 10.1055/a-2272-9794

**Published:** 2024-04-05

**Authors:** Laurelle van Tilburg, Eva P. D. Verheij, Steffi E. M. van de Ven, Sanne N. van Munster, Bas L. A. M. Weusten, Lorenza Alvarez Herrero, Wouter B. Nagengast, Erik J. Schoon, Alaa Alkhalaf, Jacques J. G. H. M. Bergman, Roos E. Pouw, Lindsey Oudijk, Sybren L. Meijer, Marnix Jansen, Michail Doukas, Arjun D. Koch, on behalf of the Dutch Barrett Expert Centers and the Dutch Barrett Expert Centers Study Group, Lodewijk A. A. Brosens, Wouter L. Curvers, Martin H. M. G. Houben, Pieter Jan F. de Jonge, Gursah Kats-Ugurlu, Jaap S. van der Laan, Ineke G. van Lijnschoten, Freek C. P. Moll, Ariadne H. A. G. Ooms, G. Mihaela Raicu, Thjon J. Tang, Jessie Westerhof

**Affiliations:** 1 Department of Gastroenterology and Hepatology, Erasmus MC Cancer Institute, University Medical Center, Rotterdam, the Netherlands; 2 Department of Gastroenterology and Hepatology, Amsterdam UMC, location University of Amsterdam, Department of Gastroenterology and Hepatology, Amsterdam Gastroenterology Endocrinology Metabolism, Cancer Center Amsterdam, Amsterdam, The Netherlands; 3 Department of Gastroenterology and Hepatology, University Medical Center Utrecht, Utrecht, the Netherlands; 4 Department of Gastroenterology and Hepatology, St. Antonius Hospital, Nieuwegein, the Netherlands; 5 Department of Gastroenterology and Hepatology, University Medical Center Groningen, University of Groningen, Groningen, the Netherlands; 6 Catharina Hospital, Catharina Cancer Institute, Department of Gastroenterology and Hepatology, Eindhoven, the Netherlands; 7 GROW School for Oncology and Developmental Biology, Maastricht University, Maastricht, the Netherlands; 8 Department of Gastroenterology and Hepatology, Isala Clinics, Zwolle, the Netherlands; 9 Department of Pathology, Erasmus MC Cancer Institute, University Medical Center, Rotterdam, the Netherlands; 10 Department of Pathology, Amsterdam University Medical Centers, Amsterdam, the Netherlands; 11 UCL Cancer Institute, University College London, London, United Kingdom; 12 Department of Pathology, University College London Hospital, London, United Kingdom; 13 Department of Pathology, University Medical Centre Utrecht, Utrecht, the Netherlands; 14 Department of Gastroenterology and Hepatology, Haga Teaching Hospital, Den Haag, the Netherlands; 15 Department of Pathology, University Medical Centre Groningen, University of Groningen, Groningen, the Netherlands; 16 Department of Pathology, Haga Teaching Hospital, Den Haag, the Netherlands; 17 Department of Pathology, PAMM, Eindhoven, the Netherlands; 18 Department of Pathology, Isala Clinics, Zwolle, the Netherlands; 19 Department of Pathology, Pathan BV, Rotterdam, the Netherlands; 20 Department of Pathology, Pathology DNA, St Antonius Hospital, Nieuwegein, the Netherlands; 21 Department of Gastroenterology and Hepatology, Ijsselland Hospital, Capelle aan den Ijssel, The Netherlands

## Abstract

**Background** This study evaluated the proportion of patients with residual neoplasia after endoscopic resection (ER) for Barrett’s neoplasia with confirmed tumor-positive vertical resection margin (R1v).

**Methods** This retrospective cohort study included patients undergoing ER for Barrett’s neoplasia with histologically documented R1v since 2008 in the Dutch Barrett Expert Centers. We defined R1v as cancer cells touching vertical resection margins and Rx as nonassessable margins. Reassessment of R1v specimens was performed by experienced pathologists until consensus was reached regarding vertical margins.

**Results** 101/110 included patients had macroscopically complete resections (17 T1a, 84 T1b), and 99/101 (98%) ER specimens were histologically reassessed, with R1v confirmed in 74 patients (75%), Rx in 16%, and R0 in 9%. Presence/absence of residual neoplasia could be assessed in 66/74 patients during endoscopic reassessment (52) and/or in the surgical resection specimen (14), and 33/66 (50%) had residual neoplasia. Residual neoplasia detected during endoscopy was always endoscopically visible and biopsies from a normal-appearing ER scar did not detect additional neoplasia. Of 25 patients who underwent endoscopic follow-up (median 37 months [interquartile range 12–50]), 4 developed local recurrence (16.0%), all detected as visible abnormalities.

**Conclusions** After ER with R1v, 50% of patients had no residual neoplasia. Histological evaluation of ER margins appears challenging, as in this study 75% of documented R1v cases were confirmed during reassessment. Endoscopic reassessment 8–12 weeks after ER seems to accurately detect residual neoplasia and can help to determine the most appropriate strategy for patients with R1v.

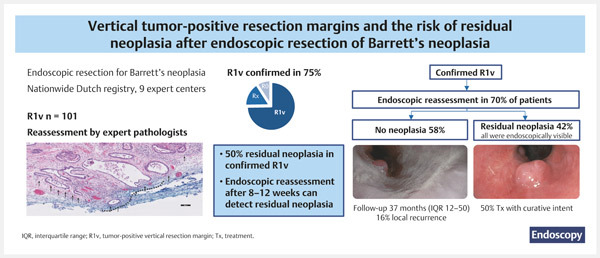

## Introduction

Endoscopic resection (ER) has become the first-line curative treatment for early neoplasia in Barrett’s esophagus (BE) because of its safety and low cancer recurrence risk during long-term follow-up 1 2 3 . Histopathological assessment of the ER specimen predicts the risks for lymph node metastasis and residual neoplasia. This assessment drives further clinical decision making, ranging from endoscopic follow-up to surgery 2 4 . Endoscopic follow-up is justified in patients with a low risk of lymph node metastasis (i. e. mucosal or superficial submucosal cancer [≤ sm1] with good to moderate tumor differentiation and no lymphovascular invasion) and a low risk of residual neoplasia, characterized by tumor-negative resection margins 1 .

Current guidelines recommend adjunct surgery in patients with tumor-positive vertical resection margins (R1v) 1 2 . However, residual neoplasia is not always present in the surgical resection specimen of patients with R1v 5 . Moreover, surgical resection is – even in high volume centers – associated with substantial mortality (0–5 %), morbidity (20 %–50 %), and decreased quality of life 6 7 8 . Surgery may thus be unwanted overtreatment in a subset of patients with documented R1v. We hypothesize that endoscopic reassessment after R1v may be able to discern patients with residual neoplasia who should be offered surgery, from those without residual neoplasia, who can be followed up endoscopically.

Published studies on residual cancer after R1v resections are scarce and review small numbers of patients 9 10 11 12 . Even though varying definitions of R1 have been used and accurate histopathological assessment of the vertical margin of ER specimens has proven to be challenging, even by experienced pathologists, these studies report lower risks of residual cancer than has generally been assumed (range 0–57 %) 9 10 11 12 . Recently, our research group reported outcomes of 138 endoscopic submucosal dissections (ESDs) performed between 2008 and 2019 in the Barrett Expert Centers in the Netherlands 5 . Vertical and/or lateral R1 resections were found in 38 ESD specimens. In 71 % of these patients, no residual cancer was present during first endoscopic reassessment, performed 8–12 weeks after ESD 5 .

Studies involving systematic reassessment of R1v margins by an experienced pathology board are currently lacking. Consequently, the risk of residual cancer following R1v resections remains unclear. This study aimed to evaluate the risk of residual neoplasia following endoscopic mucosal resection (EMR) or ESD of BE neoplasia with documented R1v. Our second aim was to report the characteristics and outcomes of R1v resections.

## Methods

This retrospective nationwide study used data from the Dutch Barrett Expert Centers registry (Netherlands Trial Register, NL7039), which has been described in detail previously 3 13 . The registry contains outcomes of all patients receiving endoscopic treatment for BE neoplasia in the Netherlands since 2008. This care is centralized in the Netherlands: endoscopists and pathologists from all nine expert centers participate in a joint training program, adhere to a unified protocol, and attend annual clinical and scientific meetings to guarantee uniform clinical management. Each Barrett Expert Center has a minimum annual caseload of 10 new patients undergoing endoscopic treatment for BE neoplasia.

The Medical Ethical Research Committee of Amsterdam University Medical Centers decided that the registry was not subject to Medical Research Involving Human Subjects and waived the need for formal ethical review and informed consent.

### Study population

All patients treated with ER for early BE neoplasia with documented R1v margins in pathology reports were included. Patients were included from January 2008 to May 2019 for EMRs and to December 2020 for ESDs. This study also included 32 ESDs with documented R1v that have been described previously 5 .

### Histopathological evaluation

ER specimens were pinned down on cork or hard wax and fixed in formalin solution for 24 hours. Specimens were then cut to 4 µm thickness at 2-mm interval for ER specimens and at 5-mm intervals for surgery specimens. Subsequently, the slides were stained with hematoxylin and eosin. Treatment with other stains (e. g. p53, desmin, pan-keratin) was performed based on the pathologist’s preference. The tumor invasion depth was classified as at least mucosal (T1a; m1-m3) or submucosal (T1b; sm1-sm3). Tumor differentiation grade was reported as well differentiated, moderately differentiated, or poorly differentiated/undifferentiated. Lymphovascular invasion was present or absent.

#### Reassessment of ER specimens

Documented pathology assessment and reassessment were performed by experienced BE pathologists. All available pathology slides of resection specimens were retrieved and up to five relevant slides regarding the vertical margin were selected by two pathologists (M.D., S.L.M.). In case of missing pathology slides or insufficient quality, formalin-fixed paraffin-embedded tissue blocks of resection specimens were retrieved and new slides were cut. In equivocal cases regarding the maximum invasion depth or vertical margin, additional slides were cut at the pathologist’s request. Relevant R1v slides were digitized for reassessment, anonymized, and stored on a secure server.

#### Vertical resection margin

The resection margins were assessed as either cancer cells unequivocally infiltrating the resection margin (R1), absence of cancer cells in the margins (R0), or nonassessable margins (Rx). All digital pathology slides were reassessed independently by one of the four participating experienced gastrointestinal pathologists (L.O., M.D., M.J., S.L.M.). The pathologists were blinded to patient, treatment, and outcomes of prior pathology assessment. Outcomes of vertical margin reassessment were compared with prior pathology reports. In case of disagreement between the vertical margin outcome of reassessment and initially documented pathology, a second pathologist blindly reassessed the slides. For cases in which the second pathologist was not in agreement with either the initial pathology report or the reassessment by the first pathologist, a consensus meeting was held with all four pathologists. In equivocal cases or in case of Rx margins, the reasons were discussed (e. g. tangential cutting, suboptimal embedding, curled lateral margins, cauterization artifacts). For confirmed R1v margins, the following characteristics were assessed: tumor width at the vertical margin (i. e. maximum width of the tumor in contact with the vertical resection margin in μm), number of R1v sites, ER specimen depth at the R1v site, and tumor differentiation at the invasive front, according to the World Health Organization classification for tumor grading 14 .

### Reassessment of surgical specimens

For patients who underwent surgery after R1v, adjunct review of the surgical specimen was performed by an experienced pathologist (L.O., M.D., S.L.M.). The presence and, if applicable, tumor stage of BE neoplasia (high grade dysplasia [HGD] or esophageal adenocarcinoma [EAC]) were reassessed to ensure all patients with residual neoplasia were detected. The presence of submucosal fibrosis, which may suggest the previous ER location, was also evaluated.

### Treatment and follow-up strategy

An ER with R1v margin is generally considered a high risk resection (i. e. noncurative). Guidelines recommend that complete staging, including positron emission tomography/computed tomography and endoscopic ultrasound to detect any lymph node metastasis or distant metastasis, should be performed before additional treatment is initiated 1 . Additional treatment, including surgery and/or chemoradiotherapy, is strongly recommended because of the presumed high risk of residual cancer 1 . The risk of lymph node metastasis is based on histopathological characteristics of the ER specimen (i. e. high risk if deep submucosal invasion [sm2 /3], poorly differentiated/undifferentiated, or with lymphovascular invasion) 1 . Patients deemed unfit or who refused surgery without signs of metastasis, were offered endoscopic follow-up. In the absence of residual neoplasia and metastasis, additional radiofrequency ablation of the residual BE segment was considered during endoscopic follow-up, to prevent possible malignant progression 3 .

### Study end points

The primary end point was presence of residual neoplasia after ER with R1v margin for BE neoplasia. Residual neoplasia was defined as the presence of HGD or EAC detected during first endoscopic reassessment within 1 cm of the ER scar or in the surgical resection specimen (see **Table 1 s** in the online-only Supplementary material). Secondary end points included: 1) outcomes of pathology reassessment of documented R1v margins; 2) accuracy of first endoscopic reassessment in detecting residual neoplasia; and 3) clinical outcomes including long-term follow-up with local recurrences. Local recurrence was defined as HGD or EAC detected during endoscopic follow-up within 1 cm of the ER scar, with at least one prior endoscopy without abnormalities.

### Statistics

Descriptive statistics were presented as means with SDs, medians with interquartile ranges (IQRs), and counts with percentages, when appropriate. Statistical analyses were performed using IBM SPSS for Windows version 25 (IBM Corp., Armonk, New York, USA). Logistic regression was used to compare outcomes among different subgroups.

## Results

### Baseline and procedure characteristics

**Fig. 1:**
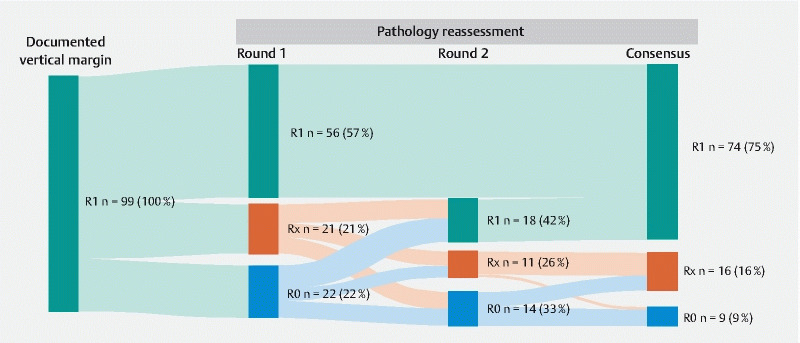
Outcomes of pathology reassessment of macroscopically complete endoscopic resection (ER) with documented tumor-positive vertical resection margins (R1v) (n = 99). Data shown as n (%) in a Sankey diagram. ER specimen with documented R1v could be retrieved for pathology reassessment in 99 /101 (98.0 %) patients with macroscopically complete resections. R1, tumor-positive vertical resection margins; Rx, nonassessable margins; R0, tumor-free resection margins.

A total of 1442 patients were treated with ER for BE neoplasia at the expert centers since 2008 ( **Fig. 1 s** ). Pathology reports showed documented R1v margins in 110 patients (7.6 %). Baseline patient, ER, and pathology characteristics are shown in Table 1 , Table 2 , and **Table 2 s** . Documented R1v was reported in 5.8 % of patients treated with EMR (73/1263) and in 20.7 % of patients treated with ESD (37/179). Most EMRs were performed in a piecemeal fashion (93.2 %), while most ESDs were en bloc resections (91.9 %). For en bloc resections (n = 39; 34 ESDs and 5 EMRs), lateral R1 margins positive for cancer were present in 14/39 patients (35.9 %). High risk characteristics for lymph node metastasis (i. e. ≥ T1sm2, poorly differentiated/undifferentiated, or with lymphovascular invasion) were present in 61.8 % of patients with documented R1v. Most procedures (n = 101; 91.8 %) were considered endoscopically successful (i. e. macroscopically complete). Macroscopically incomplete resections (n = 9) are described in supplementary **Text 1 s** and were not included in histological reassessment or evaluations for residual neoplasia.

**Table 1:** Baseline characteristics of patients with documented tumor-positive vertical resection margins (n = 110).

Patient characteristics	n = 110
Male sex	89 (80.9)
Age, mean (SD), years	69.5 (10.1)
ASA classification ≥ 3	31 (28.2)
BE length, median (IQR), cm	
Circumferential extent	2 (0–5)
Maximum extent	4 (3–7)
Paris classification (primary component)	
0-lp/Is	37 (33.6)
0-Iia	63 (57.3)
0-Iib	6 (5.5)
0-Iic	3 (2.7)
Missing	1 (0.9)
Prior treatment	6 (5.5)
ER	3 (2.7)
ER + subsequent RFA	2 (1.8)
RFA	1 (0.9)
Technique	
MBM	56 (50.9)
Cap-assisted EMR	17 (15.5)
ESD	37 (33.6)
En bloc	39 (35.5)
Piecemeal	71 (64.5)
Number of pieces	5 (4–7)
Macroscopically successful resection	101 (91.8)

ASA, American Society of Anesthesiologists; BE, Barrett’s esophagus; ER, endoscopic resection; EMR, endoscopic mucosal resection; ESD, endoscopic submucosal dissection; IQR, interquartile range; MBM, multiband mucosectomy; RFA, radiofrequency ablation. Data are n (%) unless otherwise stated.

**Table 2:** Pathology characteristics for patients with documented tumor-positive vertical resection margins following endoscopic resection for Barrett’s neoplasia (n = 110).

Documented pathology characteristics	n (%)
Maximum measured invasion depth	
T1m3	20 (18.2)
T1b	
Sm1 (< 500 µm)	37 (33.6)
Sm2/3 (≥ 500 µm)	52 (47.3)
T2 1	1 (0.9)
Differentiation grade	
Well differentiated	18 (16.4)
Moderately differentiated	50 (45.5)
Poorly differentiated/undifferentiated	42 (38.2)
Presence of lymphovascular invasion	
No	74 (67.3)
Yes	36 (32.7)
Lateral resection margins 2	
Tumor-negative (R0)	23 (59.0)
Not assessable (Rx)	2 (5.1)
Tumor-positive (R1)	14 (35.9)

1 Endoscopic submucosal dissection with partial removal of the muscularis propria containing Barrett’s neoplasia.

2 For en bloc resections only (n = 39).

### Outcomes of experienced pathologist reassessment

Pathology slides of 99/101 (98.0 %) macroscopically complete resections could be retrieved for reassessment. A median of 3 slides (range 1–5) were used for a maximum of 2 rounds of reassessment and consensus meeting by experienced pathologists ( Fig. 1 ). The presence of R1v margins was confirmed in 74.7 % of the documented and reassessed cases (74/99; 95 %CI 65.0 %–82.0 %), while the remaining vertical margins were reassessed as Rx (n = 16, 16.2 %) and R0 (n = 9, 9.1 %). R1v margins were confirmed in 90.9 % of ESDs (30/33) and in 66.7 % of EMRs (44/66) ( **Fig. 1 s** ). In patients with mucosal carcinoma, 56.3 % had confirmed R1v margins, while in patients with submucosal carcinoma, confirmed R1v was diagnosed in 78.3 %. In patients with confirmed R1v (n = 74), the median R1 tumor width at the vertical margin was 1140 µm (IQR 500–1978) ( **Table 3 s** ) and 39.2 % had more than one R1v site in the resection specimen.

**Fig. 2:**
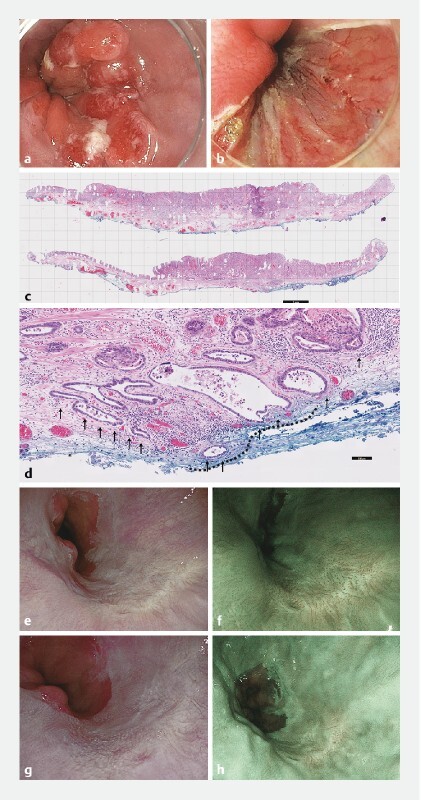
No residual neoplasia or recurrence during follow-up in a patient with confirmed tumor-positive vertical resection margin (R1v). **a** Paris type 0-Iia-Iic lesion with suspicion of submucosal invasion. **b** The lesion was resected by endoscopic submucosal dissection. **c,d** Histopathology assessment showed a well-differentiated, T1bsm1 adenocarcinoma, with lymphovascular invasion and R1v. Reassessment of the vertical margin by a panel of experienced pathologists confirmed R1v, with a width of 1500 µm cancer cells in the vertical margin (dashed line indicating vertical R1 segment and arrows indicating the invasion depth). The lymphovascular invasion is not shown. **e,f** This patient had no residual neoplasia during first endoscopic reassessment, which was confirmed with target biopsies of the resection scar. **g,h** No additional treatment was performed, and no local recurrence was detected during a follow-up of 36 months and five endoscopies.

During reassessment of 48 /99 cases of documented R1v, the pathologist could not assess the vertical margin (Rx n = 16, 16.2 %) or had some doubt regarding their assessment of radicality (n = 32, 32.3 %). Reasons preventing optimal histological assessment included tangential cutting (28.3 %), suboptimal embedding (22.2 %), curled lateral margins (15.2 %), cauterization artifacts (15.2 %), and pinning artifacts (15.2 %). Pathology images demonstrating these features that prevented optimal histopathological assessment are shown in **Fig. 2 s** and were present in 62.1 % of the EMR specimens and 21.2 % of the ESD specimens ( **Table 4 s** ).

### Findings of endoscopic reassessment

Among the patients with confirmed R1v, 52 /74 (70.3 %) underwent endoscopic reassessment after a median of 10 weeks (IQR 6–15). During first endoscopic reassessment, a visible suspicious lesion within 1 cm of the ER scar was detected in 25/52 patients (48.1 %). Of these, residual neoplasia was confirmed in 22/25 visible lesions (positive predictive value 88.0 %, Table 3 ), while no dysplasia was detected in three patients with target biopsies. These three patients underwent subsequent surgery, showing no neoplasia (n = 1), chemoradiotherapy because of lymph node metastasis (n = 1), and endoscopic follow-up over 48 months without detection of a local recurrence (n = 1). In patients without visible lesions (27/52; 51.9 %), target biopsies of the regularly healed ER scar were taken in nine patients, and did not result in additional detection of neoplasia ( Fig. 2 ). The negative predictive value of first endoscopic reassessment was 79.2 % (95 %CI 57.9 %–92.9 %) ( Table 3 ), taking into account all patients treated with subsequent surgery or undergoing endoscopic follow-up. Even though no residual neoplasia was observed, six patients were referred for subsequent surgery, which resulted in the detection of HGD in one patient and no residual neoplasia in the other patients.

**Table 3:** Predictive value of first endoscopic reassessment for the detection of residual neoplasia or local recurrence in the esophagus.

	Outcome 1		Total
	Residual neoplasia or local recurrence	No neoplasia	
First endoscopic reassessment			
Suspicious lesion	22	3	25
No lesion	5 2	19	24
Total	27	22	49

1 The absence of residual neoplasia could be confirmed in surgical resection specimens or during endoscopic follow-up. Patients without visible lesions during first endoscopic reassessment who were directly referred for chemoradiotherapy (n = 2) or had no endoscopic follow-up (n = 1) were excluded in this analysis.

2 Consisting of 1 residual neoplasia (high grade dysplasia) and 4 local recurrences detected after 7, 9, 10, and 19 months, respectively, after endoscopic resection with tumor positive vertical resection margins.

### Clinical outcomes after confirmed R1v resection

**Fig. 3:**
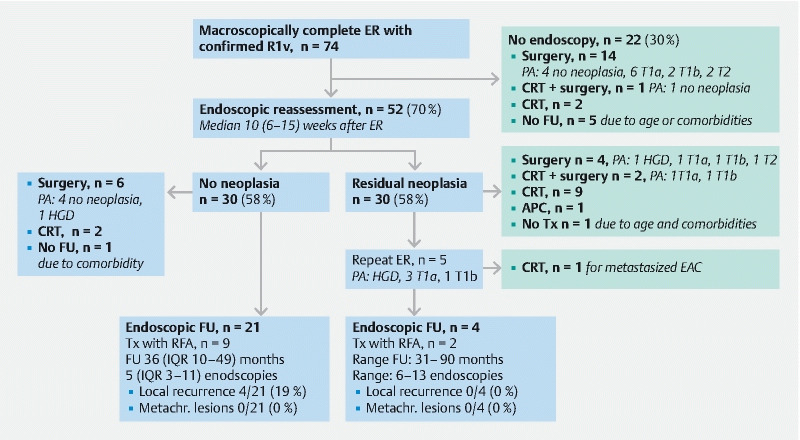
Outcomes of endoscopic resection (ER) for Barrett’s neoplasia with histological tumor-positive vertical resection margins (R1v) (n = 74). APC, argon plasma coagulation; CRT, chemo- and/or radiotherapy; EAC, esophageal adenocarcinoma; FU, follow-up; metachr, metachronous; HGD, high grade dysplasia; IQR, interquartile range; PA, pathology assessment; RFA, radiofrequency ablation; Tx, treatment.

The presence of residual neoplasia could be assessed in 66 /74 patients with confirmed R1v, of whom 50.0 % (33/66; 95 %CI 37.4 %–62.6 %) had residual neoplasia in the surgical resection specimen (n = 11) or during the first endoscopic reassessment (n = 22) ( Table 4 , Fig. 3 ). Reasons preventing surgical treatment after R1v are shown in **Table 5 s** . The tumor stages of detected residual neoplasia were HGD (n = 3), T1a (n = 10), T1b (n = 4), and ≥ T2 carcinoma (n = 16) ( Fig. 3 ). In the remaining eight patients (8/74), the presence of residual neoplasia was unknown, due to treatment with chemoradiotherapy before endoscopic reassessment (n = 2), chemoradiotherapy and surgery (n = 1), or no follow-up (n = 5).

**Table 4:** Presence of residual neoplasia after endoscopic resection of Barrett’s neoplasia according to documented tumor invasion depth and vertical margin status, as assessed by endoscopic reassessment or in the surgical resection specimen.

Invasion depth and vertical margin status	n	No residual neoplasia, n (%)	Residual neoplasia, n (%)	Could not be assessed, n 1
R1v	74	33 (50.0)	33 (50.0)	8
T1m3	9/74	6 (75.0)	2 (25.0)	1
T1sm1	28/74	10 (38.5)	16 (61.5)	2
T1sm2/3	37/74	17 (53.1)	15 (46.9)	5
Rx	16	9 (75.0)	3 (25.0)	4
R0	9	5 (62.5)	4 (44.4)	0

R1v, tumor-positive vertical resection margin; Rx, nonassessable margins; R0, tumor-free resection margins.

1 Presence of residual neoplasia could not be assessed, due to absence of endoscopic reassessment after R1v or treatment with primary chemoradiotherapy.

Residual neoplasia occurred less often after ESD (33.3 %) than after EMR (61.5 %) with confirmed R1v margin. The risk of residual neoplasia was higher, but not statistically significantly increased with increasing tumor width in the vertical margin of the ER specimen (odds ratio 1.52, 95 %CI 0.95–2.42 for every increase of 1000 µm). The specimen depth at the R1v site was limited to the muscularis mucosa in 24 patients (32.4 %), of whom 17 had submucosal carcinoma in other ER specimen parts. Of these, 14/17 underwent first endoscopic reassessment, of whom 7/14 (50.0 %) had residual neoplasia.

### Treatment of residual neoplasia

Of the patients with residual neoplasia (n = 33), 14 underwent adjunct surgery revealing HGD (n = 2), T1a (n = 7), T1b (n = 3), and T2 (n = 3) carcinoma ( Fig. 3 ). Nine patients received chemoradiotherapy after endoscopic reassessment with residual neoplasia because of a high risk of lymph node metastasis. Two patients were treated with chemoradiotherapy and surgery (T1a [n = 1] and T1b carcinoma [n = 1]) after endoscopic reassessment. In five patients, repeat ER was performed and histopathology showed HGD (n = 1), T1a (n = 3), and T1b (sm2; n = 1) EAC. Four of the patients treated with repeat ER, received endoscopic follow-up after repeat ER (range 31–90 months of follow-up with 6–13 endoscopies) and had no local recurrences or metachronous lesions. In one patient, metastasized EAC was detected shortly after repeat ER and this patient died of EAC after 7 months. Outcomes of surgical specimen reassessment are shown in **Fig. 3 s** .

### Endoscopic follow-up

In total, 25 patients received endoscopic follow-up for a median of 37 months (IQR 12–50) with 6 (IQR 3–11) endoscopies after the ER with confirmed R1v ( Fig. 3 ). Of these patients, four were previously treated with repeat ER for residual neoplasia (described above). During follow-up, four local recurrences (16.0 %) were detected within 1 cm of the ER scar after 7, 9, 10, and 19 months, respectively. These patients had T1m3 (n = 2), T1sm1 (n = 1), and Tsm2/3 (n = 1) carcinoma at baseline. Prior to detection of the local recurrence, target biopsies of the nonsuspicious ER scar were taken in 3/4 patients and showed no dysplasia. Most local recurrences (75.0 %) could be treated curatively with repeat ER (n = 2, histology HGD and T1a) and chemoradiotherapy with surgery (n = 1, histology no neoplasia). One patient with a local recurrence did not receive treatment due to the diagnosis of metastasized lung cancer. None of the patients were diagnosed with metachronous lesions, and 11/25 patients were treated with radiofrequency ablation for eradication of the residual BE epithelium.

### Outcomes of R0 and Rx diagnosis after reassessment

During reassessment by the central experienced pathologist panel, vertical margins were reassessed as Rx (n = 16) or R0 (n = 9). Among vertical Rx, presence of residual neoplasia could be assessed in 13 patients, in whom 4 (30.8 %) had residual neoplasia ( **Fig. 4 s** ). Among vertical R0, residual neoplasia was detected in four patients (44.4 %). These latter four patients had ER with lateral R1 margins (n = 2), poor tumor differentiation (n = 2), and/or lymphovascular invasion (n = 1). Residual neoplasia after Rx (n = 4) or R0 (n = 4) was treated curatively in 7 /8 patients with surgery (n = 3, histology 2 T1a), chemoradiotherapy with surgery (n = 1; histology T1a), repeat ER (n = 1, histology HGD), and repeat ER with chemoradiotherapy (n = 2, histology 1 T1a and 1 T1b). One patient was diagnosed with metastasized EAC shortly after endoscopic reassessment and died after 16 months.

## Discussion

Our results show that when histological assessment of ER specimens revealed R1v, half of the patients had no residual neoplasia afterwards. We reported on all 110 ERs with documented R1v margins that were retrospectively included in the Dutch Barrett Expert Centers registry and underwent histopathological reassessment by experienced pathologists. In 50 % of our patients with R1v confirmed by a panel of experienced pathologists, residual neoplasia was present in the surgical resection specimen or during first endoscopic reassessment. This is important, as an R1v is usually considered equal to the presence of residual cancer after ER of BE neoplasia. If residual neoplasia was present, 39 % of patients had HGD or mucosal carcinoma, which could be re-treated successfully. Residual neoplasia was accurately detected with endoscopic reassessment after 8–12 weeks.

Our findings are in line with previous studies, which have reported up to 57 % residual cancer after R1v ER 9 10 11 12 . This study provides new insights, as previous studies comprised small series or lacked reassessment by experienced pathologists. Our results confirm the apparent contradiction between a histological R1v margin after ER and absence of residual neoplasia in 50 % of the patients. The absence of residual neoplasia after R1v might be explained by: 1) ablative effects of electrocoagulation during ER; 2) compromised vascularization of the mucosal defect and effects of the immune system potentially resulting in apoptosis of cells with residual neoplasia; and 3) inaccuracy of the histological diagnosis of R1v, potentially caused by faulty endoscopy pinning, suboptimal embedding, tangential cutting, or cauterization artifacts. The latter is also reflected in the relatively large number of Rx margins (n = 16) found during reassessment. We found that most equivocal specimens revealed a combination of the aforementioned factors.

Histopathological assessment of the vertical resection margin is challenging, especially in cases of piecemeal resection. In this study, reassessment by experienced pathologists confirmed R1v in 67 % of EMRs and 91 % of ESDs. A recent study reported the concordance of different histopathological characteristics of 62 ER specimens by nine experienced pathologists 15 . Agreement among all nine pathologists regarding the vertical margin radicality was achieved in 68 % of cases 15 . In **Table 6 s** , we provide clinical recommendations for optimal handling of ER specimens to allow more accurate evaluation of vertical resection margins.

This study showed that residual neoplasia occurred more frequently after EMR (62 %) than after ESD (33 %) with confirmed R1v margin. This difference might reflect the technical aspects of ESD compared with EMR. First, during ESD, continuous submucosal lifting is performed and each separate submucosal cut is aimed underneath the lesion. At this stage, the lesion might be touched unintentionally at the submucosal side resulting in an R1 resection at the vertical margin without dissecting through tumor tissue. This will leave no tumor cells at the patient’s side of the resection. During cap-based resection (i. e. EMR), the depth of resection is less controlled and the snare takes the shortest cut while closing. The snare will cut through any tumorous tissue in its path, potentially leaving residual neoplasia at the resection base. Second, differences in patient and tumor selection between ESD and EMR may also reflect the differences in residual neoplasia after R1v.

Some limitations of this study should be addressed. The study was performed retrospectively, resulting in heterogeneous treatment and follow-up strategies. This is reflected in the relatively limited number of patients who underwent subsequent surgery after R1v given that guidelines recommend surgery in all fit patients 1 . This may be explained by increasing insights into the limited proportion of patients with residual neoplasia after R1v resections and ongoing studies assessing the potential of endoscopic follow-up in high risk patients. Follow-up strategies were not performed according to a standardized protocol, resulting in differences in timing and intervals of surveillance. Endoscopic reassessment was not available in 22 patients and biopsies were not performed in 24 patients. For piecemeal resections, lateral radicality was assessed endoscopically. This is known to be challenging, even for experienced endoscopists. Thus, plausible undocumented lateral R1 resections might partly explain cases with residual neoplasia, as repeat ER was technically feasible in some patients.

The indications for ER of BE neoplasia have been gradually expanding, resulting in more resections of high risk lesions, including submucosal EAC. This may result in an increasing rate of R1 resections in clinical care in the near future. In this study, including all documented R1v after EMR or ESD for BE neoplasia in the Netherlands, subsequent surgery often resulted in overtreatment, as no residual cancer was detected in the surgical resection specimen of 46 % of the patients referred for surgery. Additionally, no residual neoplasia was detected in 58 % of patients during endoscopic reassessment. If guidelines were followed, this would result in “unnecessary esophagectomy” in 58 % of patients; however, this is only the case in patients without signs of lymph node metastasis.

Based on previous studies and our current data, we recommend an endoscopic reassessment 8–12 weeks after ER with R1v to detect residual neoplasia and identify patients who should be referred for additional step-up treatment. Our retrospective data suggest that endoscopic assessment may be able to reliably detect residual neoplasia. In the absence of lymph node metastasis and residual neoplasia, strict endoscopic surveillance might be considered as a valid alternative strategy for patients with R1v after ER. In line with new insights on other high risk patient groups, 3-monthly endoscopic surveillance with high definition endoscopy and ultrasound (according to the PREFER study protocol, ClinicalTrials.gov Identifier NCT03222635) may be considered for patients with R1v without residual neoplasia during endoscopic reassessment. Future prospective studies with homogeneous and standardized treatment and follow-up protocols would provide evidence for an individualized approach for patients with R1v resections after ER for BE neoplasia.

In conclusion, upon confirmed vertical R1 margin after macroscopically complete ER for BE neoplasia, half of the patients had no residual neoplasia. The pathological evaluation of vertical resection margins appears challenging, especially for piecemeal resections, as only 75 % of documented R1v cases were confirmed and 16 % were re-diagnosed as Rx during reassessment. Without signs of lymph node metastasis, endoscopic reassessment can be considered after 8–12 weeks to detect residual neoplasia and decide on the most appropriate management strategy. If no abnormalities are present during first endoscopic reassessment, biopsies of the ER scar seem of limited value in detecting additional neoplasia.

## Tables 1 s–6 s, Fig. 1 s

Supplementary material Zusatzmaterial
