# Supplementary material for: Vertical tumor-positive resection margins and the risk of residual neoplasia after endoscopic resection of Barrett’s neoplasia: a nationwide cohort with pathology reassessment
Source: Endoscopy. 2024 Apr 5;56(8):559–68. doi: 10.1055/a-2272-9794 (PMC11288659; doi:10.1055/a-2272-9794)
Supplement: Supplementary file 1 — Supplementary material [file 23475supmat_10-1055-a-2272-9794.pdf]

Supplementary material

Vertical tumor-positive resection margins and the risk of residual neoplasia after endoscopic resection of Barrett's neoplasia: a nationwide cohort with pathology reassessment

Laurelle van Tilburg*, **Eva PD Verheij***, Steffi EM van de Ven, Sanne N van Munster, Bas LAM Weusten, Lorenza Alvarez Herrero, Wouter B Nagengast, Erik J Schoon, Alaa Alkhalaf[8], Jacques JGHM Bergman, Roos E Pouw, Lindsey Oudijk, Sybren L Meijer, Marnix Jansen, Michail Doukas*, Arjun D Koch*, on behalf of the Dutch Barrett Expert Centers and the Dutch Barrett Expert Centers study group.

* Shared first/last authorship; both authors contributed equally to this publication.

Dutch Barrett Expert Centers study group: Lodewijk AA Brosens, Wouter L Curvers, Martin HMG Houben, Pieter Jan F de Jonge, Gursah Kats-Ugurlu, Jaap S van der Laan, Ineke G van Lijnschoten, Freek CP Moll, Ariadne HAG Ooms, G Mihaela Raicu, Thjon J Tang and Jessie Westerhof

Supplementary material

Table 1s Definitions.

Characteristics	Definition
**BE neoplasia**	Low grade dysplasia, high grade dysplasia, or esophageal adenocarcinoma located in a Barrett's esophagus.
**Endoscopic follow-up**	All endoscopies performed after the ER with tumor-positive vertical resection margin, excluding endoscopic dilatations.
**Endoscopic reassessment**	The first endoscopy after ER with tumor-positive vertical resection margin during which the scar of the ER was assessed for residual neoplasia.
**Local recurrence**	After ER, the patient had at least one endoscopy with a non-suspicious ER scar and no BE neoplasia during histopathology assessment (if applicable) AND<br>1) Presence of a visible lesion within 1cm of the ER scar with suspicion of high grade dysplasia or esophageal adenocarcinoma detected during endoscopic follow-up OR<br>2) Absence of a visible lesion during endoscopic follow-up but histopathology within 1cm of the ER scar showing high grade dysplasia or esophageal adenocarcinoma.
**Metachronous lesions**	Development of high grade dysplasia or esophageal adenocarcinoma in the residual BE segment, at least >1cm from the ER scar.
**Residual neoplasia**	1) Presence of a visible lesion within 1cm of the ER scar with suspicion of high grade dysplasia or esophageal adenocarcinoma detected during first endoscopic reassessment<br>2) Absence of a visible lesion during first endoscopic reassessment but histopathology within 1cm of the ER scar showing high grade dysplasia or esophageal adenocarcinoma<br>3) Presence of high grade dysplasia or esophageal adenocarcinoma detected in the surgical resection specimen performed within 6 months after ER with R1v margin.
**Vertical margin tumor-positive (R1v)**	Presence of cancer cells in the vertical (i.e. deep) ER margin, i.e. an irradical resection.
**Vertical margin not assessable (Rx)**	Not assessable vertical ER margin, due to endoscopy and/or histopathological factors.
**Vertical margin tumor-negative (R0)**	Absence of cancer cells in the vertical ER margin. A radical resection.
**Visible lesion**	Abnormality with suspicion for BE neoplasia detected during endoscopy.

BE, Barrett's esophagus; ER, endoscopic resection.

Supplementary material

Table 2s Baseline documented pathology characteristics of EMR and ESD (n=110).

	Total n=110	EMR n=73	ESD n=37
**Maximum measured invasion depth**			
T1m3	20 (18.2%)	16 (21.9%)	4 (10.8%)
T1b			
Sm1 (<500 microns)	37 (33.6%)	28 (38.4%)	9 (24.3%)
Sm2/3 (≥ 500 microns)	52 (47.3%)	29 (39.7%)	23 (62.2%)
T2[1]	1 (0.9%)	0	1 (2.7%)
**Differentiation grade**			
G1	18 (16.4%)	14 (19.2%)	4 (10.8%)
G2	50 (45.5%)	30 (41.4%)	20 (54.1%)
G3/4	42 (38.2%)	29 (39.8%)	13 (35.1%)
**Presence of LVI**			
No	74 (67.3%)	53 (72.6%)	21 (56.8%)
Yes	36 (32.7%)	20 (27.4%)	16 (43.2%)
**Lateral resection margins[2]**			
Tumor-negative (R0)	23 (59.0%)	1 (20.0%)	22 (64.7%)
Not assessable (Rx)	2 (5.1%)	1 (1.4%)	1 (2.9%)
Tumor-positive (R1)	14 (35.9%)	3 (60.0%)	11 (32.4%)

Data presented as n with %, median (IQR) or mean with SD, according to the nature of the data. R1 defined as cancer cells present in the resection margin, Rx defined as not assessable margins, R0 defined as absence of cancers cells in the resection margin. [1]Endoscopic submucosal resection with partial removal of the muscularis propria containing BE neoplasia. [2]For en bloc resections only. Abbreviations: EMR, endoscopic mucosal resection; ESD, endoscopic submucosal dissection; LVI, lymphovascular invasion MBM, multiband mucosectomy.

Supplementary material

Table 3s Additional histopathological characteristics of macroscopic complete ER with confirmed R1v during reassessment (n=74).

**Characteristics**	**n=74**
**Tumor width in the vertical margin in µm (IQR)**	1140 (500-1978)
**Number of R1v sites**	
1	45 (60.8%)
2	18 (24.3%)
3	8 (10.8%)
4	3 (4.1%)
**Differentiation grade at the invasive front**	
G1	20 (27.0%)
G2	41 (55.4%)
G3/4	13 (17.6%)
**ER specimen depth at R1v**	
Mucosa	0
Muscularis mucosa	24 (32.4%)
Submucosa	50 (67.6%)

Data presented as n with % or median (IQR), according to the nature of the data. ER, endoscopic resection; R1v defined as cancer cells in the vertical resection margin.

Supplementary material

Table 4s Reasons preventing accurate pathology assessment of the vertical resection margin of the ER specimen.

Characteristic	Total<br>n=99	EMR<br>n=66	ESD<br>n=33
≥1 reason preventing accurate pathology assessment of the vertical resection margin	48 (48.5%)	41 (62.1%)	7 (21.2%)
Tangential cutting	28 (28.3%)	26 (39.4%)	2 (6.1%)
Suboptimal embedding	22 (22.2%)	21 (31.8%)	1 (3.0%)
Curled lateral resection margin	15 (15.2%)	14 (21.2%)	1 (3.0%)
Cauterization artifact	15 (15.2%)	13 (19.7%)	2 (6.1%)
Pinning artifact	15 (15.2%)	13 (19.7%)	2 (6.1%)
Superficial or irregular extending specimen	5 (5.1%)	5 (7.6%)	0
Fragmentation	4 (4.0%)	3 (4.5%)	1 (3.0%)

Data presented as n with %. EMR, endoscopic mucosal resection; ER, endoscopic resection; ESD, endoscopic submucosal dissection.

Supplementary material

Table 5s Reasons preventing subsequent surgery in patients with macroscopic complete ER with confirmed R1v during reassessment (n=47).

**Characteristic**	**Total**<br>n=47
Patients unfit for surgery	28 (59.6%)
Due to comorbidities	27 (57.4%)
Due to advanced age	8 (17.0%)
Patient wish	18 (38.3%)
Considered low-risk EAC (i.e. absence of risk factors for lymph node metastasis)	2 (4.3%)

Data presented as n with %. More than one reason preventing surgery can be present per patient.

Supplementary material

Table 6s Clinical recommendations for optimal handling of endoscopic resections of Barrett's neoplasia.

Clinical recommendation	Purpose and findings in the current study
In case of piecemeal resection, the completeness of the resection at the lateral margin should be determined by the endoscopist.	To prevent residual cancer or local recurrence located at the lateral resection margins.
The ER specimen should include a sufficient amount of submucosa.	To prevent vertical R1 resections of Barrett's neoplasia.<br>In this study, the specimen depth at the R1v site was limited to the muscularis mucosa in 24 patients, of whom 17/24 patients had BE neoplasia invading the submucosa in other parts of the same ER specimen.
The ER specimen should be pinned on a hard surface (e.g. on cork) with the mucosal side up, preferably performed by the endoscopist directly after ER.	Immediate pinning and fixation of the ER specimen allows for adequate orientation and tissue preservation (size and shape) to prevent curling of the lateral borders and shrinkage.<br>In this study, curling of the lateral margins prevented accurate pathology assessment of the vertical margin in 15/99 cases.
Overstretching by pinning down the ER specimen should be avoided.	To prevent tears in the ER specimen.
The pins should preferably not perforate Barrett's neoplasia and especially the area with suspicion of the deepest tumor invasion should be avoided.	To prevent artifacts and allow for accurate assessment of the resection margin(s).<br>In this study, a needle mark was present at the potential location of the vertical R1 resection in 15/99 cases.
Photographs of the ER specimen should be taken directly after pinning down.	For adequate orientation with mapping of the lesion and margins in order to compare the macroscopic appearance with endoscopy findings.
The vertical margin (and for en-bloc lateral margins) should be inked.	

ER, endoscopic resection; R1, irradical resection, i.e. tumor cells infiltrating the resection margin; R1v, tumor-positive vertical resection specimen

Supplementary material

Text 1s Outcomes of macroscopically incomplete resections.

The majority of procedures (n=101; 91.8%) were considered endoscopically successful (i.e. macroscopically complete resections). The remaining procedures (n=9; 6 EMRs and 3 ESDs) were macroscopically incomplete due to severe fibrosis and/or deep invasion. In 8 of these 9 patients (88.9%), residual neoplasia was confirmed and could be treated with additional surgery (n=4; revealing T1a (n=1), T2 (n=1), and T3 carcinoma (n=2)), CRT (n=1), or palliative care (n=3)). In the remaining patient with a macroscopically incomplete ER (PA T2), no residual neoplasia was detected during the first endoscopic reassessment; a T2 local recurrence was detected after 33 months of endoscopic follow-up with 8 endoscopies for which palliative radiotherapy was offered due to advanced age and comorbidities.

Supplementary material

Fig. 1s Flowchart of patient inclusion and outcome of histopathological assessment of the vertical resection margin.

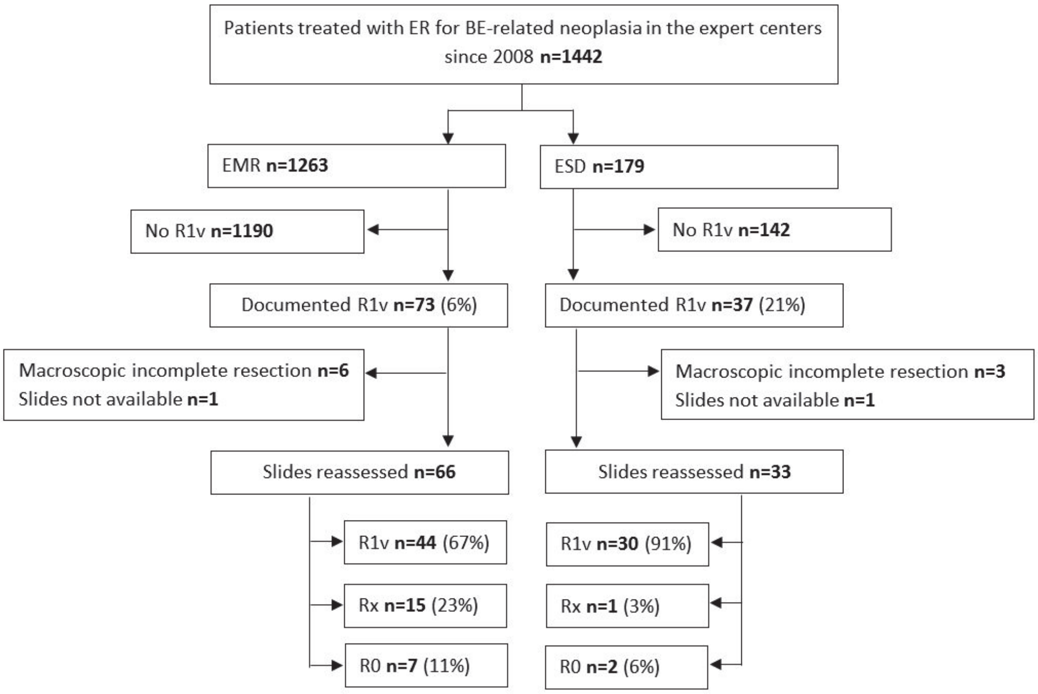

BE, Barrett's esophagus; EMR, endoscopic mucosal resection; ER, endoscopic resection; ESD, endoscopic submucosal dissection; R1v, tumor-positive vertical resection margin defined as cancers cells in the vertical resection margin; Rx, not assessable vertical resection margin; R0, tumor-negative vertical resection margin.

Supplementary material

Fig. 2s Images of pathology slides with reasons preventing optimal histopathological assessment of vertical resection margins after endoscopic resection of BE neoplasia; curled margin (A), suboptimal embedding (B), tangential cutting (C), cauterization artifacts (D) and fragmentation (E).

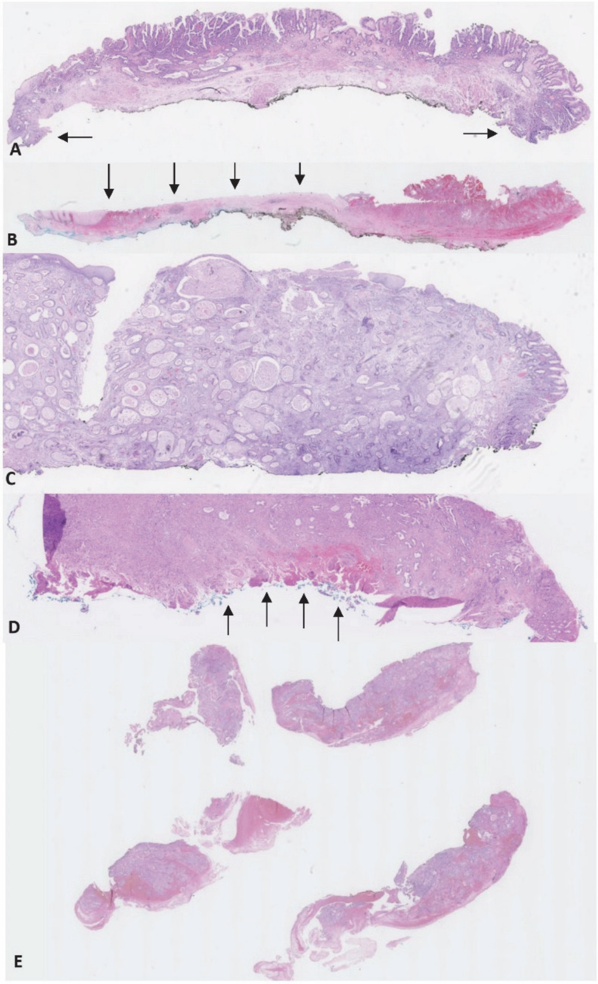

Supplementary material

Fig. 3s Outcomes of pathology reassessment of patients treated with additional surgery after a macroscopic complete ER with documented R1v (n=37*), either directly after R1v resection or after endoscopic reassessment.

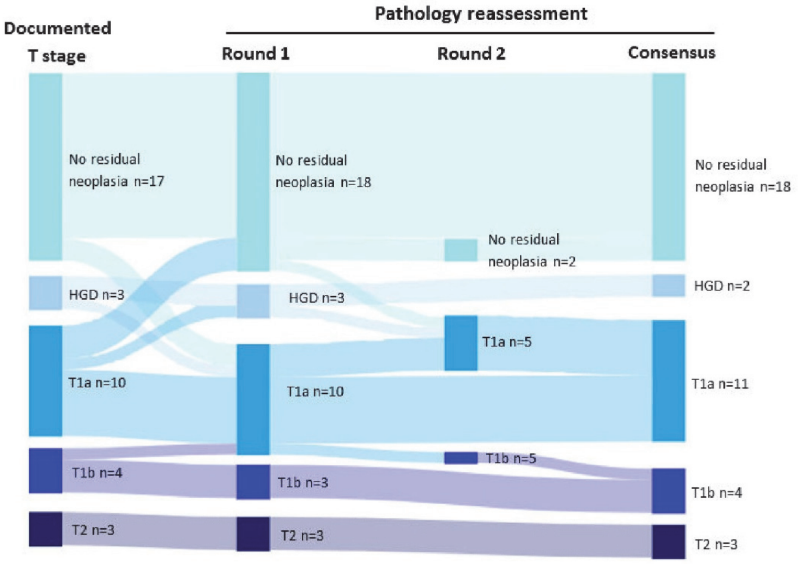

Data shown as n with % in a Sankey diagram. *The esophagectomy specimens could be retrieved for pathology reassessment in 37/39 patients. ER, endoscopic resection.

Supplementary material

Fig. 4s Outcomes of ER for BE neoplasia with Rx (n=16) or R0 (n=9) vertical margin during pathology reassessment.

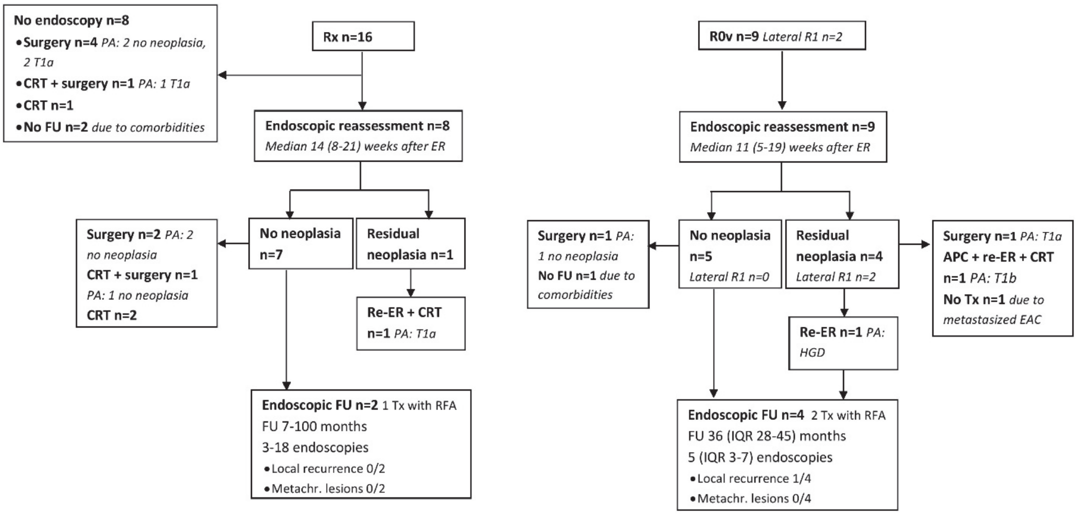

BE, Barrett's esophagus, CRT, chemo- and/or radiotherapy; EAC, esophageal adenocarcinoma; ER, endoscopic resection; FU, follow-up; metachr., metachronous; PA, pathology assessment; RFA, radio frequent ablation; Tx, treatment. The local recurrence after R0 resection was treated with re-ER (PA: T1a carcinoma).
